# Speciation of Tetracycline and Its Zn^2**+**^ Complexes in Aqueous Solution and Their Antiproliferative Activity in Non-Small Lung Cancer Cells

**DOI:** 10.1021/acsomega.6c04056

**Published:** 2026-07-17

**Authors:** Chiara Abate, Federica Carnamucio, Claudia Foti, Marsela Ceraj, Massimiliano Cordaro, Sandro R. P. da Rocha, Ottavia Giuffrè

**Affiliations:** † Dipartimento di Scienze Chimiche, Biologiche, Farmaceutiche ed Ambientali, 18980 Università di Messina, Viale F. Stagno d’Alcontres 31, Messina 98166, Italy; ‡ Department of Pharmaceutics and Center for Pharmaceutical Engineering and Sciences - School of Pharmacy, 6889 Virginia Commonwealth University, 410 N 12th St, Richmond, Virginia 23298, United States

## Abstract

Tetracycline (TC), the primary member of the tetracyclines, is a widely used broad-spectrum antibiotic that has also attracted interest for its nonantibiotic biological effects, including anticancer activity. The aim of this study is to assess the acid–base behavior of TC, its ability to form complexes with Zn^2+^ under various conditions, and to demonstrate that such species enhance the antitumor activity of TC in non-small cells lung cancer (NSCLC). The speciation study on TC and its Zn^2+^ complexes was performed in aqueous NaCl solutions at different temperatures (15 ≤ t/ °C ≤ 45) and I = 0.15 mol L^–1^ via potentiometric, UV–vis spectrophotometric, and ^1^H NMR titrations. The protonation and formation constants of the species were calculated, and their temperature dependence was evaluated, along with the sequestration capacity of TC toward Zn^2+^. The speciation results were essential to identify the most suitable Zn^2+^/TC ratio and experimental conditions to ensure significant complex formation for the in vitro cytotoxicity studies in A549 cells. The biological results showed that Zn^2+^ complexation significantly affected the cytotoxicity of TC, enhancing its antiproliferative activity compared to the free ligand.

## Graphical abstract

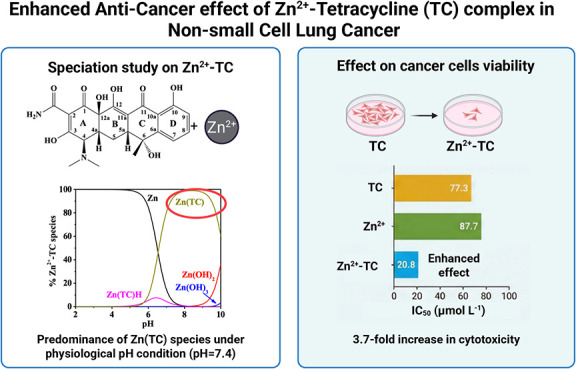

## 1 Introduction

Tetracycline (TC, Figure 1) has been widely used as a feed additive to promote livestock growth and as a broad-spectrum antibiotic in the treatment of human and animal diseases since 1948, owing to its low cost, chemical stability, and the absence of relevant adverse side effects.^1–4^ It is the primary natural member of tetracyclines (TCs), the first antimicrobials discovered nearly 70 years ago by B. Duggar, after whom the class is named.^5,6^ TCs exert their activity by inhibiting protein synthesis in both Gram-positive and Gram-negative bacteria, blocking the binding of the aminoacyl-tRNA to bacterial ribosomes. This inhibition reduces intestinal bacterial populations and limits competition for nutrient absorption.^4,5,7^ However, their widespread employment over the past 40 years has significantly contributed to the emergence of bacterial resistance.^3,8,9^ To address this global health challenge, numerous semisynthetic TCs have been developed.^10–12^ Despite the rise in resistance, TCs are still employed in the treatment of various bacterial infections, including urinary, gastrointestinal, ocular, and respiratory diseases, as well as chlamydial infections, acne, periodontitis, pneumonia, rosacea, rheumatoid arthritis, and malaria prophylaxis.^7,10,13^ Beyond their antimicrobial action, TCs exhibit anti-inflammatory and antitumor properties, with evidence demonstrating their ability to enhance antitumor T-cell responses and support bone-targeted therapies for metastatic cancers.^14–18^

**Figure 1:**
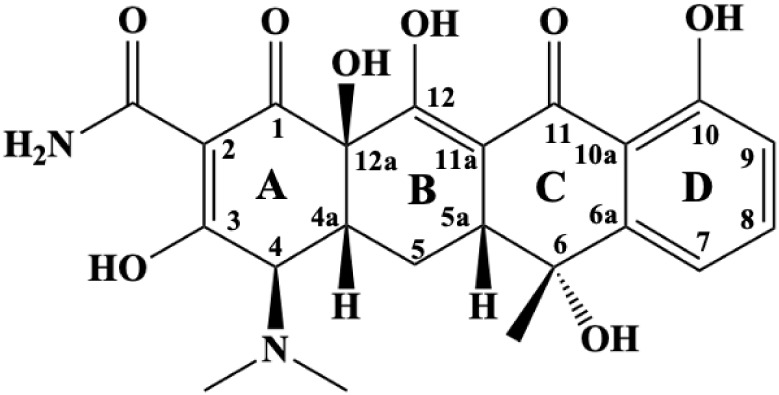
Chemical structure of (4S,4aS,5aS,6S,12aS)-4-(Dimethylamino)-3,6,10,12,12a-pentahydroxy-6-methyl-1,11-dioxo-1,4,4a,5,5a,6,11,12a-octahydro-2-tetracencarboxamide (tetracycline, TC).

Structurally, TCs consist of a tetracyclic naphthacene-carboxamide scaffold comprising four fused six-membered rings, with molecular weights ranging from 442 to 586 g mol^–1^. This rigid polycyclic framework provides substantial chemical stability, which corroborates their global use in the treatment of human and animal diseases, as well as in industry, agriculture, and aquaculture.^2,5–7,19^ Key groups, including the dimethylamino one at C4, the α-stereochemistry at ring junctions C4a and C12a, and the β-ketoenol moiety at C10, C11, and C12 positions, are believed to be crucial for antibacterial activity.^5^ Structural modifications at these positions typically diminish antimicrobial efficacy while enhancing nonantibiotic biological effects.^5,10^ TCs display acid–base behavior due to the presence of carboxamide, dimethylamine, hydroxyl, and keto groups.^7^ Variations in protonation state, depending on the pH, modulate solubility, physicochemical properties, metal-binding capacity, and ultimately biological function.^5^ Indeed, the presence in the chemical structure of several potential binding sites, such as O- and N-groups, allows the formation of complex species with various metal cations, which strongly influence the pharmacological and biochemical reactivity of TCs.^1,5,20,21^

In the specific case of TC, in recent years, complex formation with metal ions has attracted increasing attention. For instance, some studies have focused on the TC complexation with Ca^2+^, Mg^2+^, Cu^2+^, and Al^3+^.^10,13,22–24^ In this study, the attention was focused on Zn^2+^, which is one of the most abundant essential metals in the human body, with outstanding regulatory, structural, and catalytic functions, as well as antioxidant, anti-inflammatory, and chelating abilities.^25,26^ It is also a potential therapeutic agent for various human diseases.^25,27^ In biological fluids, Zn^2+^ complexes have also played anticancer roles with good selectivity and low toxicity.^28^ Moreover, extracellular Zn levels modulate the bioavailability of various ligands and the binding affinity of their corresponding receptors, affecting the initiation of intracellular signaling pathways.^25^

In light of the above, the evaluation of the influence of the complexation of TC with Zn^2+^ on its antitumor activity is of great importance for potential applications in medicine. Therefore, the object of this study is TC, the primary member of TCs, chosen as a key reference for its widespread use as a drug for human and animal diseases, its cost-effectiveness, commercial availability, broad antibacterial spectrum, and chemical stability. This speciation study concerns TC and its complexes with Zn^2+^ in NaCl aqueous solutions over a wide temperature range (15 ≤ t/ °C ≤ 45, at I = 0.15 mol L^–1^) by different analytical techniques (potentiometry, UV–vis and ^1^H NMR spectroscopy) and an in vitro study on the cytotoxicity of TC and Zn^2+^-TC complexes in NSCLC cells.

## 2 Experimental Section

### 2.1 Chemicals

Tetracycline hydrochloride (TC) solutions were prepared by weighing and dissolving the commercial product (Alfa Aesar, 96.0%). Zinc­(II) chloride (Sigma-Aldrich, ≥ 98.0%) was weighed, and the solution was standardized by EDTA (Fluka, ≥ 99.0%) titrations. NaOH (Fluka, 32.0%) and HCl (Sigma-Aldrich) solutions, used for UV–vis spectrophotometric and potentiometric measurements, were prepared by dilution and standardized with potassium acid phthalate (Sigma-Aldrich, ≥ 99.5%) and sodium carbonate (Fluka, puriss. p.a. ≥ 99.5%), respectively, predried in an oven (110 °C, ∼1 h). NaCl solutions were prepared by direct weighing of the respective salt (Sigma-Aldrich, ≥ 99.0%), predried in an oven (110 °C, ∼1 h). Distilled water (conductivity < 0.1 μS cm^–1^) and class A glassware were used for all solutions.

### 2.2 UV–Vis Equipment and Procedure

A Varian Cary 60 spectrophotometer equipped with a fiber optic probe with a 1 cm path length was used to perform titrations. The instrument was interfaced to a PC by the Varian Cary WinUV (version 5.3) software, and a Metrohm glass electrode (Ross type 8102, from Thermo/Orion) was connected, in turn, to a Metrohm 713 potentiometer in order to simultaneously record the data of absorbance (A) and pH vs volume of titrant (mL) for each titration point. The titrant was delivered in a thermostated cell by means of a Metrohm 665 Dosimat, while N_2_ was bubbled in the aqueous solutions under constant magnetic stirring to avoid both CO_2_ and O_2_. For the acid–base behavior of TC, 20 mL of solutions (0.03 ≤ C_TC_/ mmol L^–1^ ≤ 0.1), HCl (0.12 ≤ C_H_ /mmol L^–1^≤ 0.4) and NaCl (0.15 mol L^–1^) were titrated with standard NaOH in the pH range 3.0–11.0. For the study of speciation related to Zn^2+^–TC system, 20 mL of solutions containing the ligand (0.05 ≤ C_TC_/ mmol L^–1^ ≤ 0.1) and metal (0.025 ≤ C_M_/ mmol L^–1^ ≤ 0.05) at different ratios (M:L = 1:1, 1:2, 1:3), HCl (0.2 ≤ C_H_ /mmol L^–1^ ≤ 0.4), and NaCl (0.15 mol L^–1^) were titrated with standard NaOH in the pH range of 3.0–11.0. Both protonation and formation constants were determined at t = 15, 25, 37, and 45 °C. The spectra were obtained by scanning from 205 to 500 nm, and a baseline containing only HCl, NaCl, and H_2_O in the same conditions of ionic strength (I = 0.15 mol L^–1^) was recorded before each measurement to consider the contribution of the matrix. The details of the experimental conditions of the titrations are reported in Table S1 of Supporting Information.

### 2.3 Potentiometric Equipment and Procedure

A Metrohm-Titrando 809 automated potentiometer, equipped with a combined glass electrode ORION (type Ross 8102SC) and a Metrohm Dosino 800 automatic dispenser, was used to perform potentiometric measurements as titrations. The instrument was interfaced with a PC by Metrohm TIAMO 2.0 software that acquires experimental data (mL/mV), tracks the emf stability, and monitors other parameters, including titrant delivery and data acquisition. The estimated accuracy of the system is ±0.15 mV and ±0.002 mL for emf and titrant volume readings, respectively. The potentiometric titrations were carried out in thermostated cells connected to a D1-G Haake thermostated, consisting of adding standard NaOH to 20 mL of solutions containing the metal, Zn^2+^ (1 ≤ C_Zn_/ mmol L^–1^ ≤ 2), ligand (1 ≤ C_TC_/ mmol L^–1^ ≤ 4), at different concentration ratios (1:1, 1:2, 1:3), HCl (3 ≤ C_H_/ mmol L^–1^ ≤ 12), and NaCl (0.15 mol L^–1^). The titrations were performed under different conditions of temperature (15 ≤ t/ °C ≤ 45) while constant magnetic stirring and nitrogen bubbling occurring in the cell, ensuring homogeneity of the solutions in the absence of O_2_ and CO_2_ interference. The details of the experimental conditions of the potentiometric titrations are given in Table S1 of Supporting Information. Furthermore, for each measurement, independent titrations of HCl with the addition of standard NaOH were performed under the same experimental conditions of temperature and ionic strength (I = 0.15 mol L^–1^), to determine the standard electrode potential (E^0^) and pK_w_ values.

### 2.4 ^1^H NMR Equipment and Procedure

The ^1^H NMR spectra were recorded using a Varian 500 MHz VNMRJNMR spectrometer. The chemical shift values were obtained with reference to the dioxane peak as an internal standard (δ_CH_dioxane_ = 3.75 ppm), and the coupling constants, J, in Hz. The analyses were conducted on aqueous solutions with a coaxial tube (d_6__acetone) and to reduce the water signal, the presaturation technique was adopted at 25 °C. The titrations were initially performed in 20 mL of NaCl solution (I = 0.15 mol L^–1^) containing TC (C_TC_ = 2 mmol L^–1^) and HCl (C_H_ = 6 mmol L^–1^) varying the pH by adding standard NaOH in the pH range of 2.3–11.0. Subsequently, the titrations were performed in 25 mL of NaCl solution (I = 0.15 mol L^–1^) containing TC (C_TC_ = 2 mmol L^–1^), Zn^2+^ (C_Zn_ = 2 mmol L^–1^) and HCl (C_H_ = 6 mmol L^–1^), while adding standard NaOH in the pH range of 2.2–5.8. The experimental conditions of the titrations are reported in Table S1 of Supporting Information.

### 2.5 Cell Culture

A549 cells were seeded at a density of 1 × 10^4^ cells/mL in 25 cm^2^ cell culture flasks and maintained in Dulbecco’s Modified Eagle Medium (DMEM) supplemented with sodium pyruvate, 10% fetal bovine serum (FBS), and 1% penicillin–streptomycin (PS). Cells were incubated at 37 °C in a Thermo Scientific CO_2_ incubator under a humidified atmosphere containing 5% CO_2_. The culture medium was replaced every 2 days. Cells were passaged when they reached approximately 80–90% confluency.

### 2.6 A549 Cell Viability

Cells were seeded in 96-well plates at a density of 1 × 10^5^ cells per well and allowed to adhere and grow for 24 h in DMEM supplemented with 10% FBS and 1% PS at 37 °C in a humidified atmosphere containing 5% CO_2_. Following incubation, treatment solutions containing TC, Zn^2+^–TC complexes, or free Zn^2+^ were added to the wells at concentrations ranging from 0.1 μmol L^–1^ to 500 μmol L^–1^. After treatment, cells were incubated for an additional 24 h under the same conditions. Cell viability was determined using the MTT assay (3-(4,5-dimethylthiazol-2-yl)-2,5-diphenyltetrazolium bromide; Invitrogen, Thermo Fisher Scientific). Briefly, cells were washed twice with phosphate buffer saline (PBS), and 110 μL of 1.09 mmol L^–1^ MTT solution (prepared in PBS) was added to each well. Plates were incubated for 4 h at 37 °C in 5% CO_2_. Subsequently, 80 μL of the solution was removed from each well, and 55 μL of dimethyl sulfoxide (DMSO) was added to dissolve the formazan crystals. Plates were then covered and incubated for an additional 10 min at 37 °C. Absorbance was measured at 540 nm using a multimode microplate reader (Synergy H1, BioTek).

### 2.7 Post-Processing Calculations

The UV–vis data of spectrophotometric titrations were processed by the HypSpec program,^29^ enabling the refinement of molar absorption coefficient values (ε) of all species, and protonation and formation constants. Experimental data of potentiometric titrations were processed using the BSTAC4 and STACO4 programs. In addition to formation constants, these programs enable the determination of other parameters, such as the analytical concentrations of the reagent, E^0^ and junction potential. The most trustworthy speciation model for the systems was obtained considering the resulting standard and mean deviations, the formation percentage of the species, and the simplicity of the model. The dependence of formation constant values on temperature was determined using the LIANA program. More details on these software are provided in the ref.^30^ The ^1^H NMR data were processed using HypNMR2008,^31^ enabling the determination of formation constants and the individual chemical shift values of the species, calculated by analyzing the observed signals and the fast mutual exchange on the NMR time scale. Finally, the speciation diagrams and the formation ion percentages of the species were provided using the Hyss program.^32^

## 3 Results and Discussion

### 3.1 Acid–Base Properties of Tetracycline

The acid–base behavior of TC was investigated in aqueous NaCl solutions at I = 0.15 mol L^–1^, over a wide temperature range (15 ≤ t/°C ≤ 45), by first performing spectrophotometric titrations, followed by ^1^H NMR to compare and confirm the results at t = 25 °C. The UV–vis spectra, shown in Figure 2a, vary significantly with pH. The peaks in the region between 250 and 400 nm are linked to the π→π* and n→π* transitions of the TC chromophore groups. Around 275–280 nm at acidic pH values, the peaks are more defined and intense. However, at basic pH, the peaks are less intense and have a broader shape. Around 350–370 nm, a bathochromic shift of the absorption maximum occurs toward longer wavelengths as the pH increases.

**Figure 2:**
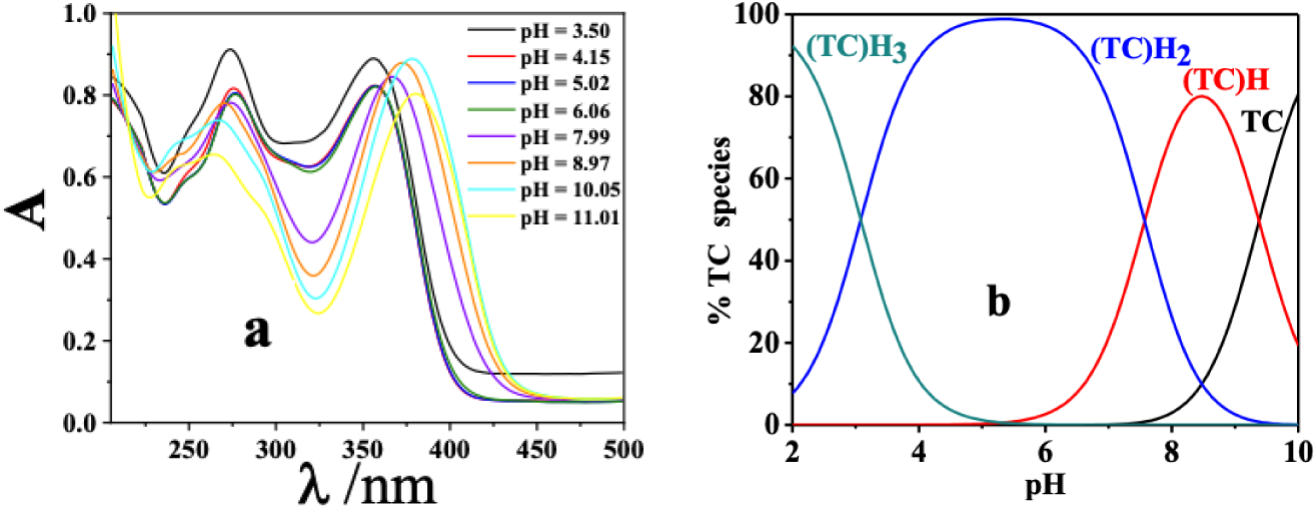
**a-b.** (a) UV–vis spectra of TC at selected pH; (b) speciation diagram of TC species. Conditions: t = 37 °C, I = 0.15 mol L^–1^, C_TC_= 0.05 mmol L^–1^.

As is known, TC is a polyfunctional molecule containing multiple protonation sites, including the dimethylamino group (position C(4)) and three hydroxyl moieties (−OH, positions C(3), C(10), and C(12)) (Figure 1). The presence of all these distinct functional groups results in a complex protonation pattern, reflected by the formation of three successive species: (TC)­H, (TC)­H_2_, and (TC)­H_3_. As reported by *Zhao et al.*, the first step (LH) likely involves the protonation of the alcoholic group at C(3), while the second and third protonation sites correspond to the alcoholic groups at C(10) or C(12) and the dimethylamine at C(4), respectively.^33^ The protonation constant values, under all of the investigated conditions, are reported in Table 1. As is notable, increasing the temperature leads to a slight decrease in log K values.

**Table 1:** Protonation Constant Values of TC Obtained via UV-Vis Spectrophotometric Titrations under Various Temperature Conditions in NaCl, *I* = 0.15 mol L ^–1^

Species	t = 15**°**C	t = 25**°**C	t = 37**°**C	t = 45**°**C
**log**β^a^				
TC)H	9.52(4)^b^	9.46(1)^b^	9.38(2)^b^	9.15(3)^b^
(TC)H_2_	17.40(5)	17.10(1)	16.85(3)	16.43(4)
(TC)H_3_	20.63(1)	20.77(3)	20.03(2)	19.43(2)
**logK** ^c^				
(TC)H	9.52	9.46	9.38	9.15
(TC)H_2_	7.88	7.64	7.47	7.28
(TC)H_3_	3.23	3.67	3.18	3.00

a **Reaction:** TC + rH = (TC)­H_r_, charges omitted for simplicity.

b ≥95% confidence interval.

c **Reaction:** (TC)­H_r‑1_ + H = (TC)­H_r_, charges omitted for simplicity.

Protonation constants reported here are fairly consistent with the literature data. Under physiological conditions (t = 37 °C, I = 0.15 mol L^–1^ in NaCl), the literature values are logβ = 9.052, 16.323, and 19.485 for (TC)­H, (TC)­H_2_, and (TC)­H_3_,^34^ respectively, which are fairly close to the values reported here in Table 1. The speciation diagram at 37 °C (Figure 2b) highlights that around physiological pH (7.4), 50% of TC exists as a zwitterionic species, (TC)­H_2_^0^. This amphoteric behavior may be crucial for its biological activity, as neutral species more readily cross biological barriers, affecting membrane permeability and pharmacokinetics.^35^

A comparison between spectrophotometric and ^1^H NMR data (Table 2) demonstrates good agreement between different techniques, confirming the robustness of the adopted speciation model.

**Table 2:** Comparison of Protonation Constant Values of TC Obtained via UV-Vis Spectrophotometric and ^1^H NMR Titrations in NaCl, I = 0.15 mol L^–1^, t = 25 °C

logβ^a^			
Species	UV–vis	^1^H NMR	Suggested
(TC)H	9.46(1)^b^	9.34(4)^b^	9.40(4)^b^
(TC)H_2_	17.10(1)	17.01(9)	17.06(9)
(TC)H_3_	20.77(3)	20.1(1)	20.4(1)

a **Reaction:** TC + rH = (TC)­H_r_, charges omitted for simplicity.

b ≥95% confidence interval.

Moreover, analysis of the ^1^H NMR data demonstrates that the chemical shifts of signals in TC are sensitive to pH variations, as highlighted in Figure 3. Protons on the phenolic D ring exhibit moderate chemical-shift changes (Δδ ≈ 0.2–0.3 ppm). In contrast, the C(6) hydroxyl proton shows a pronounced downfield shift of nearly 1.2 ppm, while the N(4) dimethylaminomethyl protons display substantial variations exceeding 0.7 ppm. These observations are consistent with the presence of a protonated dimethylamino group (ammonium form) in TC. Upon increasing pH, this ammonium proton is neutralized first, followed sequentially by deprotonation of the less acidic hydroxyl and enolic protons. The ^1^H NMR spectrum of TC recorded in H_2_O (Figure S1, Supporting Information) exhibits well-resolved resonances corresponding to distinct structural motifs. Signals from aromatic ring D include a triplet at 7.51 ppm (H8), a doublet at 7.13 ppm (H7), and a doublet at 6.91 ppm (H9). In the aliphatic region, the C(6) hydroxyl proton appears as a singlet at 3.96 ppm. A multiplet at 3.02 ppm is assigned to H4a, while a singlet at 2.91 ppm corresponds to the six equivalent methyl protons of the dimethylamino substituent at C4. The C5 methylene protons appear as multiplets at 2.17 ppm (Hα) and 1.75 ppm (Hβ), reflecting their enantiotopic nature. Additionally, the C6 methyl group is observed as a singlet at 1.53 ppm. Notably, the H4 proton is not detected at pH 2.3, likely due to rapid exchange or line broadening under strongly acidic conditions. The H5a signal is presumed to be obscured by overlap with the residual acetone signal in the 2.96–2.92 ppm region. Complementary ^1^H NMR analysis in deuterated methanol (CD_3_OD) (Figure S2, Supporting Information) enables clearer observation of exchangeable acidic protons associated with hydroxyl and amide functionalities, as highlighted in the magnified inset. Overall, TC exhibits well-defined polyprotic behavior, with thermodynamic stability strongly influenced by temperature. This acid–base framework is essential for understanding its subsequent coordination chemistry with Zn^2+^.

**Figure 3:**
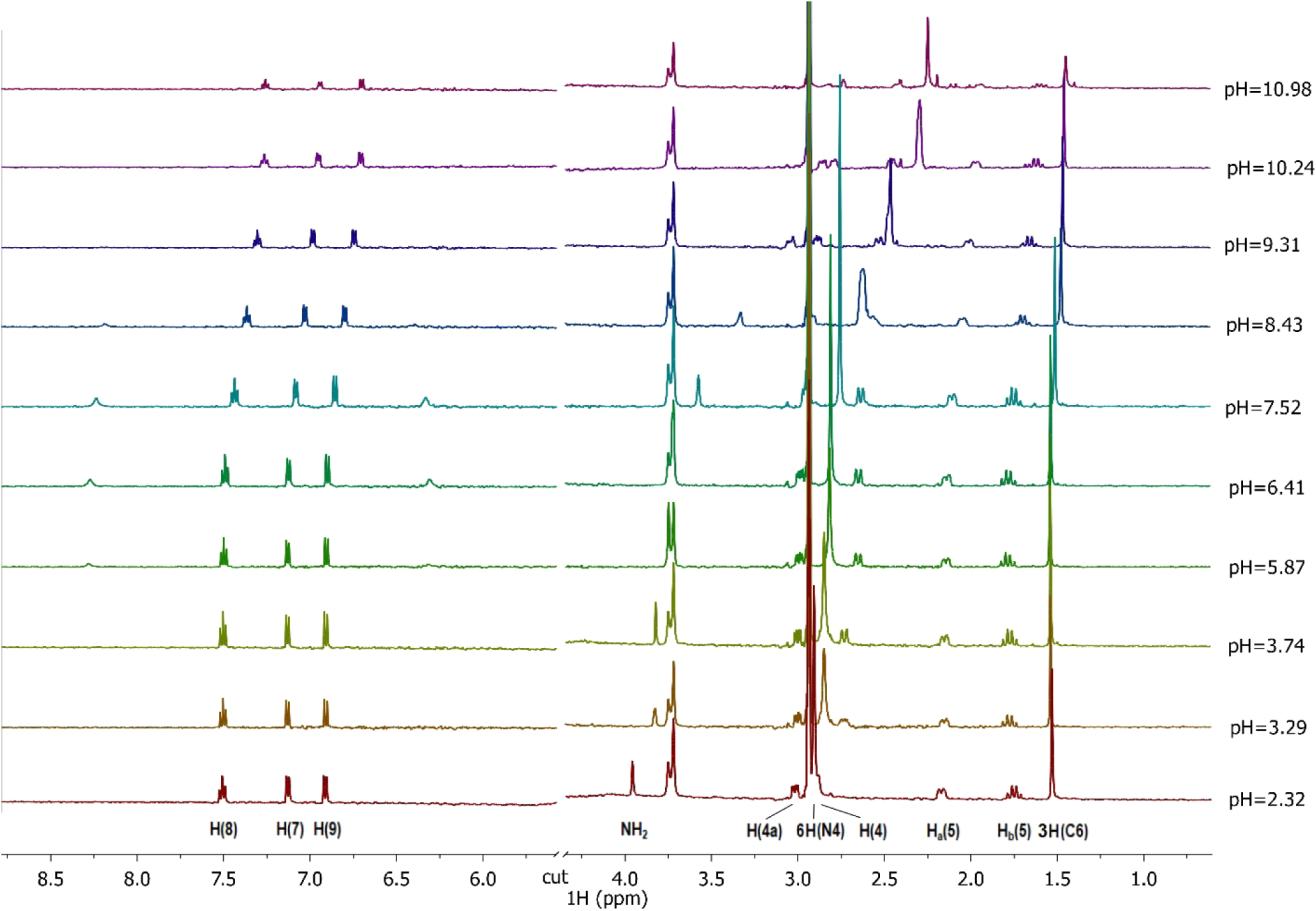
Superimposed ^1^H NMR spectra of TC at various pH, C_TC_ = 2 mmol L^–1^, t = 25 °C.

### 3.2 Interaction of TC with Zn^2**+**^ in Aqueous Solution

Once the acid–base behavior of TC had been established, its interaction with Zn^2+^ was investigated in aqueous NaCl solutions at I = 0.15 mol L^–1^ and 15 ≤ t/°C ≤ 45 by potentiometric titrations. The hydrolytic formation constant values of Zn^2+^ species are reported in Supporting Information (Table S2). As shown in Table 3, TC forms three main species with Zn^2+^, namely Zn­(TC)­H_2_, Zn­(TC)­H, and Zn­(TC). As observed for the protonation equilibria, an overall decrease in complex stability is detected with increasing temperature. The comparison between the speciation diagrams obtained at 25 and 37 °C is reported in Figure 4. Under the potentiometric experimental conditions, precipitation was observed at pH ≥ 6.0, likely due to the increased contribution of hydrolyzed Zn^2+^ species at higher pH values. As shown in the diagram of Figure 4, at strongly acidic conditions (pH < 3.0), the metal exists almost exclusively as free Zn^2+^, accounting for nearly 100% of the total metal concentration. As the pH increases, the formation of protonated complex species is observed. The Zn­(TC)­H_2_ species begins to form at pH ≈ 3.0 and reaches its maximum formation percentage (20%) at pH ≈ 4.5 and t = 25 °C. The Zn­(TC)H species is visible at pH > 4.5, increasing its formation at higher temperatures with a maximum peak at pH ≈ 5.8. At pH > 5.5, the Zn­(TC) complex becomes the dominant species, mainly at 37 °C.

**Table 3:** Formation Constant Values of Zn^2+^–TC under Various Temperature Conditions Obtained via Potentiometric Titrations in NaCl, I = 0.15 mol L^–1^

**Species**	t = 15**°**C	t = 25**°**C	t = 37**°**C	t = 45**°**C
logβ^a^				
Zn(TC)H_2_	19.28(10)^b^	19.07(7)^b^	18.05(10)^b^	18.26(8)^b^
Zn(TC)H	14.39(5)	13.61(7)	13.87(3)	13.40(9)
Zn(TC)	8.55(3)	7.90(6)	8.08(2)	8.42(2)
**logK** ^c^				
Zn(TC)H_2_	4.89	5.46	4.18	4.86
Zn(TC)H	5.84	5.71	5.79	4.98
Zn(TC)	8.55	7.90	8.08	8.42

a **Reaction:** M + TC + rH = M­(TC)­H_r_, charges omitted for simplicity.

b ≥95% confidence interval.

c **Reaction:** M + (TC)­H_r-1_ + rH = M­(TC)­H_r_, charges omitted for simplicity. ≥95% confidence interval.

**Figure 4:**
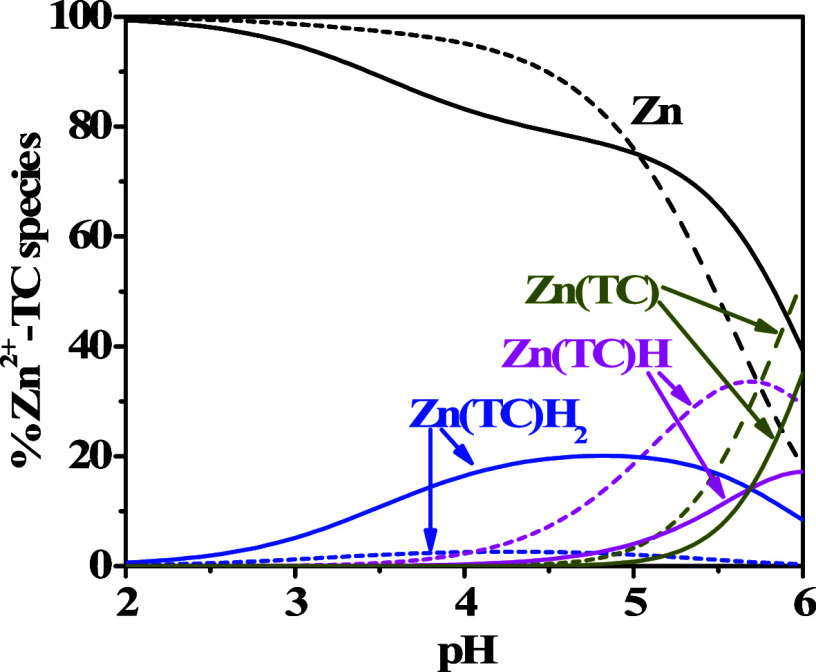
Speciation diagram of Zn^2+^–TC at C_Zn_ = 1 mmol L^–1^, C_TC_ = 2 mmol L^–1^, I = 0.15 mol L^–1^. Dashed lines refer to t = 37 °C, straight lines refer to t = 25 °C.

Literature data on TC complexes with Zn^2+^ are few and dated.^36^ Anyway, formation constants for the Zn^2+^–L complexes obtained here under physiological conditions are in fairly good agreement with previously reported literature data. Specifically, Brion et al.^37^ obtained a speciation model with four species, namely Zn­(TC)­H_2_, Zn­(TC)­H, Zn­(TC)_2_H_2_, and Zn­(TC)_2_H_3_, where the stability constants of the Zn­(TC)­H_2_ and Zn­(TC)H species at t = 37 °C and I = 0.15 mol L^–1^ in NaCl are logβ = 18.245 and 13.522, respectively, which differ by approximately 0.2 and 0.35 log units from the values reported here (18.05 and 13.87, respectively), indicating a fairly good consistency between the two data sets. In the experimental conditions adopted for this study, no species with two TC molecules is formed in significant amounts. Notably, no literature data are available for the formation constant of the Zn­(TC) species under physiological conditions (t = 37 °C and I = 0.15 mol L^–1^ in NaCl). Overall, the reported results provide a comprehensive thermodynamic picture compared to the limited literature data currently available on Zn^2+^–TC interactions in aqueous solutions.

UV–vis spectrophotometric titrations were subsequently carried out to validate the speciation model, to extend the investigated pH range, and to confirm the formation constant values obtained by potentiometric measurements. This technique enables experiments to be performed at significantly lower metal and ligand concentrations, closer to those relevant in real biological systems (C_max_ of TC in plasma is 0.01 mmol L^–1^),^38^ thus preventing the precipitation phenomena observed under potentiometric conditions. However, under lower concentrations, the Zn­(TC)­H_2_ species was formed only to a limited extent, resulting in spectral variations that were insufficient for a reliable refinement of its formation constant via UV–vis data. All formation constant values obtained via spectrophotometric analyses are reported in Table 4. Despite this limitation, the consistency between the formation constants obtained for the remaining complexes confirms the robustness of the proposed speciation model. The speciation diagram under the concentrations used for the spectrophotometric titrations is shown in Figure 5. Under these conditions, the Zn­(TC)­H_2_ species is not very significant, and at pH > 7, most of the TC is present as the Zn­(TC) species.

**Table 4:** Formation Constant Values of Zn^2+^–TC Species under Various Temperatures Obtained via Spectrophotometric Titrations in NaCl, I = 0.15 mol L^–1^

Species	t = 15**°**C	t = 25**°**C	t = 37**°**C	t = 45**°**C
logβ^a^				
Zn(TC)H_2_	19.28	19.07	18.05	18.26
Zn(TC)H	14.39	13.87	14.2(1)^b^	13.40
Zn(TC)	8.68(2)^b^	7.93(1)^b^	8.42(3)	7.65(3)^b^
**logK** ^c^				
Zn(TC)H_2_	4.89	5.20	3.85	4.86
Zn(TC)H	5.71	5.94	5.8	5.75
Zn(TC)	8.68	7.93	8.42	7.65

a **Reaction:** M + TC + rH = M­(TC)­H_r_, charges omitted for simplicity.

b ≥95% confidence interval.

c **Reaction:** M + (TC)­H_r‑1_ + rH = M­(TC)­H_r_, charges omitted for simplicity.

**Figure 5:**
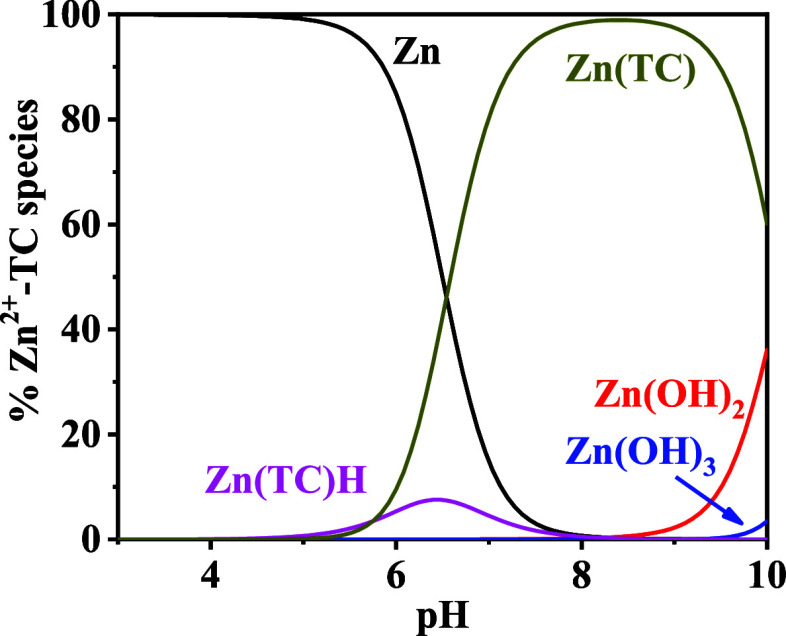
Speciation diagram of Zn^2+^–TC species at C_Zn_ = 0.025 mmol L^–1^, C_TC_ = 0.05 mmol L^–1^, I = 0.15 mol L^–1^, t = 37 °C.

^1^H NMR spectroscopy provided additional validation of the thermodynamic results and yielded formation constant values at t = 25 °C that are in good agreement with those obtained by potentiometric and spectrophotometric techniques (Table 5), further supporting the reliability of the proposed speciation model. ^1^H NMR analyses of Zn^2+^–TC solutions were conducted under the same experimental conditions adopted for the free ligand. Figure 6 shows ^1^H NMR spectra of Zn^2+^–TC at various pH levels. Titrations above pH 5.8 were not feasible due to the formation of a precipitate, likely attributable to hydrolytic species of Zn^2+^.

**Table 5:** Comparison of Formation Constant Values of Zn^2+^–TC at t = 25 °C and I = 0.15 mol L^–1^ in NaCl Obtained via Potentiometry, UV-Vis, and ^1^H-NMR Titrations

**logβ** ^a^				
Species	Potentiometry	^1^H NMR	UV–vis	Suggested
Zn(TC)H_2_	19.07(7)^b^	19.5(4)^b^	19.07^b^	19.3(4)^b^
Zn(TC)H	13.61(7)	13.61	13.61	13.61(7)
Zn(TC)	7.90(6)	7.90	7.93(1)	7.92(6)

a **Reaction:** L + rH = LH_r_, charges omitted for simplicity.

b ≥95% confidence interval.

**Figure 6:**
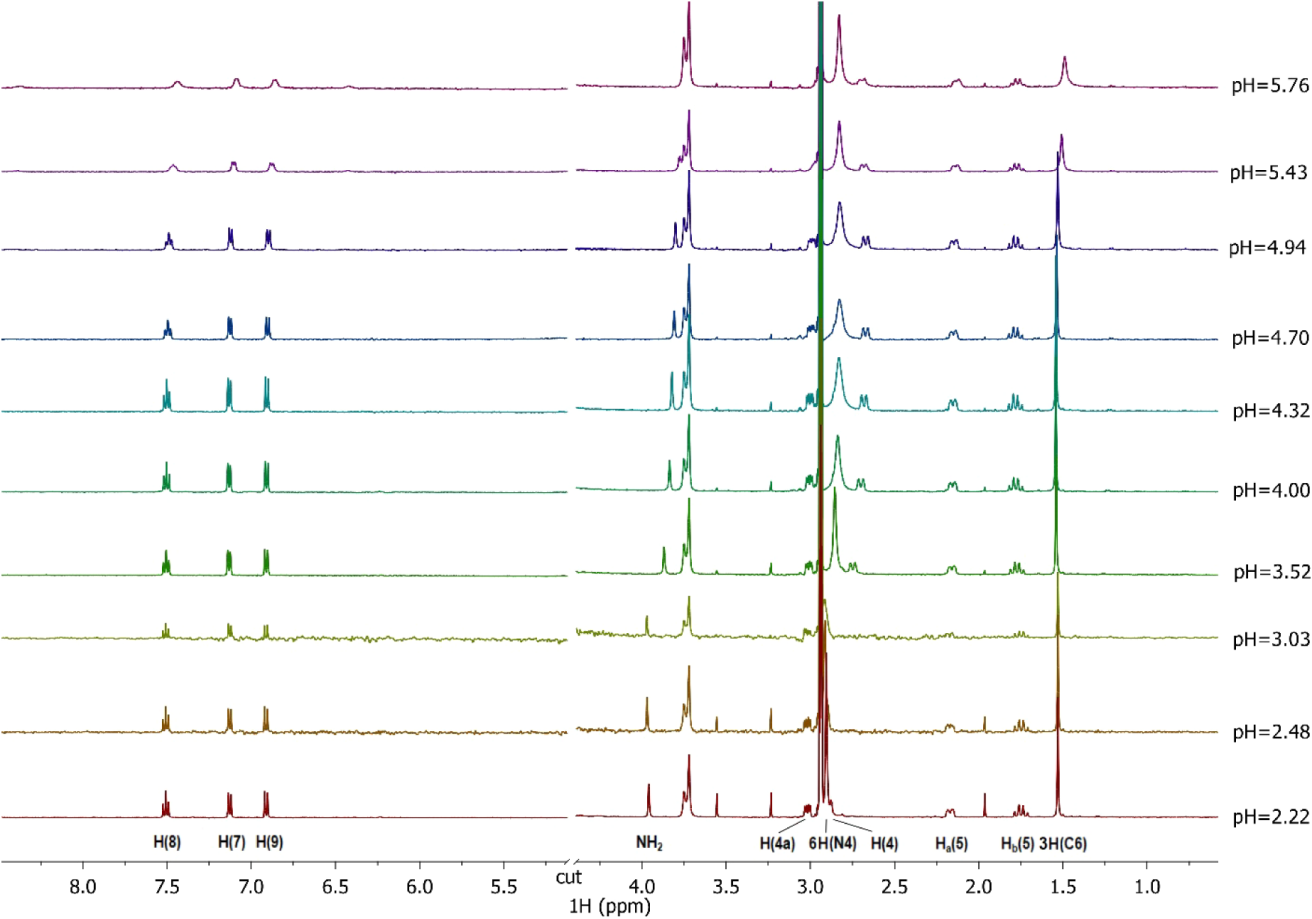
Superimposed ^1^H NMR spectra of Zn^2+^–TC at various pH, C_Zn_ = C_TC_ = 2 mmol L^–1^, t = 25 °C.

Analysis of the pH dependence of the ^1^H NMR chemical shifts shows that all proton environments are affected by pH variations. In detail, the aromatic protons of the phenolic ring exhibit moderate chemical-shift changes (Δδ ≈ 0.2–0.3 ppm), whereas the C(6) hydroxyl proton undergoes a pronounced shift of nearly 1.2 ppm. The dimethylamino methyl protons display variations exceeding 0.7 ppm, confirming that TC exists predominantly in the protonated ammonium form at low pH. Upon increasing pH, this ammonium proton is neutralized first, followed by deprotonation of the less acidic hydroxyl and enolic groups. In the presence of Zn^2+^, comparison between the chemical shifts of free L vs Zn^2+^-L reveals systematic variations that provide insight into the coordination mode. The most significant changes involve the dimethylamino methyl protons and the aromatic protons of the phenolic ring, indicating that the N(4) amino group and the C(10) phenolic oxygen are the primary sites involved in Zn^2+^ coordination within the investigated pH range. Other proton signals display more limited variations, which can be attributed to indirect electronic effects induced by complex formation. Comparison charts of the chemical shift signals of free TC and TC in the presence of Zn^2+^ vs pH have also been reported in Supporting Information (Figure S3). Overall, the ^1^H NMR results are fully consistent with the thermodynamic data and corroborate the speciation model derived from potentiometric and spectrophotometric analyses. This is confirmed by the perfect superposition of the calculated chemical shifts with the experimental ones (Figure S4 of Supporting Information). The values of the calculated chemical shifts for each TC (L) nucleus are reported in the Supporting Information (Table S3).

### 3.3 Dependence of Protonation and Formation Constant Values on Temperature

The dependence of the protonation and formation constants of TC and Zn^2+^–TC complexes on temperature was investigated by applying the van’t Hoff equation, as already done in other studies:^39,40^

$$\log^{T}\beta ={\log\beta }_{\theta }+\Delta H_{\theta }\left(\frac{1}{\theta }-\frac{1}{T}\right)$$

where log^T^β is the equilibrium constant at temperature T (K), θ is the temperature reference value of 298.15 K, and logβ_θ_ is the equilibrium constant at the reference temperature, ΔH^0^ is the standard enthalpy change, and R is the gas constant. The resulting values, calculated at 25 °C and I = 0.15 mol L^–1^, are summarized in Table 6.

**Table 6:** Thermodynamic Parameters of TC and Zn^2+^-TC Species at t = 25 °C and I = 0.15 mol L^–1^

Species	111ΔG/kJ mol^‑1^ ^a^	1ΔH/kJ mol^‑1^ ^a^	TΔS/kJ mol^‑1^ ^a^
(TC)H	–53.7	–21(4)^b^	33(4)^b^
(TC)H_2_	–43.7	–33(4)	11(4)
(TC)H_3_	–19	–14(4)	5(4)
Zn(TC)H_2_	–12.2	–31(10)	–19(10)
Zn(TC)H	–24.0	–26(9)	2(9)
Zn(TC)	–45.2	–22(9)	23(9)

a Reactions: (TC)­H_r‑1_ + rH = (TC)­H_r_ and M + (TC)­H_r‑1_ + rH = M­(TC)­H_r_; charges omitted for simplicity.

b ≥95% confidence interval.

For the protonation equilibria of TC, all reactions are characterized by exothermic enthalpy changes (ΔH = −21, −33, −14 kJ mol^–1^). The entropy contributions show a different behavior: protonation leading to LH is associated with a significant positive TΔ*S* term (33 kJ mol^–1^), while progressively lower values are observed for (TC)­H_2_ and (TC)­H_3_ (11 and 5 kJ mol^–1^, respectively). This indicates that the first protonation step benefits from a substantial entropy gain, likely related to solvent reorganization and charge compensation effects, whereas subsequent protonation steps are progressively less favored entropically. The driving forces of the formation equilibria of Zn^2+^–TC complexes differ among the species. The formation of Zn­(TC)­H_2_ and Zn­(TC)­H is enthalpy-driven (ΔH = −31 kJ mol^–1^, TΔ*S*= −19 kJ mol^–1^ for Zn­(TC)­H_2_; ΔH = −26 kJ mol^–1^, TΔ*S*= 2 kJ mol^–1^ for Zn­(TC)­H). For the fully deprotonated Zn­(TC) species, the enthalpic and entropic contributions are similar in absolute value (ΔH = −22 kJ mol^–1^ and TΔS= 23 kJ mol^–1^).

### 3.4 Sequestering Ability

The effective ability of TC (L) to sequester Zn^2+^ under different conditions was evaluated by means of the pL_0.5_ parameter, which represents the cologarithm of the total ligand concentration required to bind 50% of the metal ion present in solution. It provides a comprehensive measure of metal-binding efficiency, accounting for all competing equilibria. The sequestering ability was quantified at pH = 7.4, I = 0.15 mol L^–1^ in NaCl, and at different temperatures (15 ≤ t/°C ≤ 45). The pL_0.5_ values were calculated using the Boltzmann-type equation,^41,42^

$$\chi =\frac{1}{1+10^{\left(\mathrm{pL}-{\mathrm{pL}}_{0.5}\right)}}$$

where χ is the sum of the molar fractions of all Zn^2+^–TC complex species and pL is the cologarithm of the total ligand concentration. Figure 7 provides a graphical comparison of the sequestering ability at different temperatures. The results indicate a marked temperature dependence of the Zn^2+^ sequestration efficiency. At 25 °C, a pL_0.5_ value of 2.12 was obtained, corresponding to a low affinity of TC for Zn^2+^ at physiological pH. An increase in temperature leads to enhanced sequestering ability, with pL_0.5_ values increasing to 3.00 at 37 °C and to 3.35 at 45 °C. At 15 °C, a lower pL_0.5_ value (2.80) is observed, indicating less efficient metal binding at lower temperatures. This trend reflects the combined effect of temperature on formation constants and speciation equilibria, as higher temperatures favor the formation of complexed Zn^2+^ species under physiological conditions. Overall, the pL_0.5_ analysis demonstrates that TC exhibits improved Zn^2+^ sequestration at temperatures close to or above physiological values, highlighting the relevance of temperature effects when evaluating metal–ligand interactions in biologically relevant environments.

**Figure 7:**
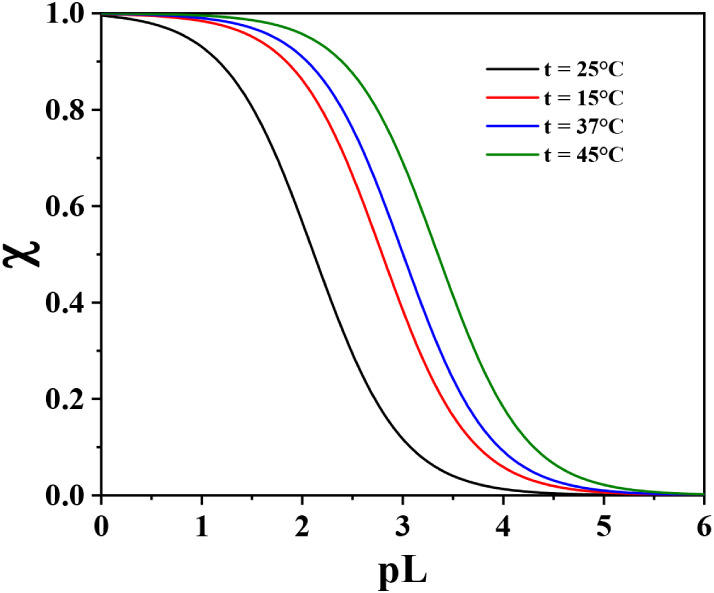
Comparison of the sequestering ability of TC (L) against Zn^2+^ under different temperature conditions at pH = 7.4, I = 0.15 mol L^–1^ in NaCl.

### 3.5 In Vitro Cytotoxicity of Zn^2+^–TC Complex

TC, beyond its well-known antimicrobial activity, has garnered increased attention for its potential anticancer properties. Recent studies have demonstrated that TC can enhance antitumor immune responses and improve cytotoxic T-cell activity in tumors, including NSCLC models.^16^ Moreover, clinical studies suggest that TC administration may improve outcomes in NSCLC patients receiving targeted therapies such as EGFR tyrosine kinase inhibitors.^17^ In parallel, Zn^2+^ is essential in numerous cellular processes, and increased intracellular levels have been shown to inhibit proliferation and induce apoptosis in NSCLC cells.^43^ In this context, the effect of Zn^2+^ coordination on the antiproliferative activity of TC was evaluated in adenocarcinomic NSCLC. Prior to biological evaluation, the speciation study allowed to identify the most appropriate drug-to-metal ratio under physiological conditions and to ensure significant formation of the Zn^2+^–TC complex. The analysis indicated that a 1:2 Zn^2+^:TC ratio resulted in high formation percentages of the Zn­(TC) species, as shown in Figure 5. Based on these findings, the biological assays were conducted under conditions favoring the predominance of the complex species. Dose–response curves of A549 cell viability after 24 h treatment with TC, Zn^2+^ and the Zn^2+^–TC complex are shown in Figure 8.

**Figure 8:**
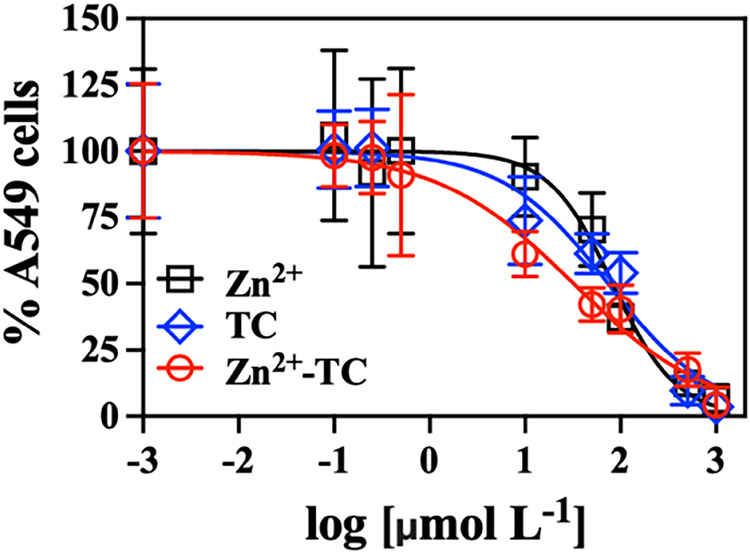
Dose–response curves of A549 cell viability after 24 h treatment with TC, Zn^2+^, and the Zn^2+^–TC complex. Data are expressed as mean ± standard deviation (SD) (n = 6) and fitted using nonlinear regression. The calculated IC_50_ values were 20.8 μmol L^–1^ for Zn–TC (95% confidence interval: 4.9–34.3 μmol L^–1^), 87.7 μmol L^–1^ for Zn^2+^ (95% confidence interval: 48.6–86.7 μmol L^–1^), and 77.3 μmol L^–1^ for TC (95% confidence interval : 47.7–102.0 μmol L^–1^). Statistical analysis was performed using one-way ANOVA followed by Tukey’s multiple comparisons test. Significant differences were observed between Zn^2+^–TC and TC (p = 0.0090) and between Zn^2+^-TC and Zn^2+^ (p = 0.0092), whereas no significant difference was found between Zn^2+^ and TC (p = 0.2352).

In the present study, TC alone exhibited an IC_50_ value of 77.3 μmol L^–1^ against A549 cells. To the best of our knowledge, no studies have reported the IC_50_ of TC in A549 cells. However, related TC derivatives have shown a broad range of antiproliferative activities in NSCLC models. For instance, doxycycline demonstrated the ability to strongly inhibit A549 cell growth, with IC_50_ values of 1.06 μmol L^–1^ after 48 h of treatment.^44^ On the other hand, minocycline exhibited a considerably higher IC_50_ value of approximately 218.6 μmol L^–1^ in A549 cells after 24 h.^45^ Therefore, the IC_50_ value obtained for TC in the present study indicates moderate intrinsic antiproliferative activity against A549 cells.

Our experiments also showed an IC_50_ value of 87.7 μmol L^–1^ for Zn^2+^. Similarly, previous studies demonstrated that Zn^2+^ treatment at concentrations between 50 and 100 μmol L^–1^ significantly reduced A549 cell viability and promoted apoptosis-related responses, indicating cytotoxic effects within the same concentration range observed in the present study.^43^ In addition, Zn^2+^-containing tetracycline complexes have also demonstrated promising anticancer activity.^46^ Recently, a novel Zn^2+^-Minocycline complex was reported to exhibit potent antiproliferative activity against H460 lung cancer cells, reducing cell viability to 7.98% at a concentration of 100 μg mL^–1^.^47^ El-Megharbel et al. also suggested that the enhanced activity of Zn^2+^-Minocycline was associated with the coordination of Minocycline to Zn^2+^ through oxygen donor groups, which may improve cellular interactions and biological activity.^47^

Interestingly, in this study, the Zn^2+^–TC complex displayed a markedly lower IC_50_ value of 20.8 μmol L^–1^, corresponding to an approximately 3.7-fold reduction compared to free TC and a 4.2-fold reduction compared to Zn^2+^ alone, demonstrating that Zn^2+^ coordination significantly potentiated the antiproliferative activity of TC. Therefore, these findings support the potential of the Zn^2+^–TC complex as a promising anticancer candidate against NSCLC.

## 4 Conclusions

This study provides a comprehensive speciation study of TC and its interaction with Zn^2+^ in aqueous solutions at I = 0.15 mol L^–1^ over a wide temperature range (15 ≤ t/ °C ≤ 45). The acid–base behavior of TC was characterized by three well-defined protonation steps, confirming its polyprotic nature and highlighting the influence of temperature on protonation equilibria. Complexation studies revealed the formation of three main Zn^2+^–TC species, namely Zn­(TC)­H_2_, Zn­(TC)­H, and Zn­(TC), whose stability constants were consistently determined by potentiometric, UV–Vis, and ^1^H NMR techniques. The agreement among these independent methods supports the reliability of the proposed speciation model. Under near-physiological conditions, the Zn­(TC) species is predominant, and ^1^H NMR data indicate that coordination mainly involves the dimethylamino group and the phenolic oxygen of TC. Thermodynamic analysis showed that both enthalpic and entropic contributions govern complex formation, with their relative importance depending on the protonation state of the ligand. The sequestering ability of TC toward Zn^2+^ increases with temperature, indicating enhanced metal binding under biologically relevant conditions. Importantly, biological assays demonstrated that Zn^2+^ coordination significantly enhances the antiproliferative activity of TC in A549 cells, with a marked reduction in IC_50_ compared to the free drug. This result highlights a synergistic effect between TC and Zn^2+^ and suggests that metal complexation can be an effective strategy to modulate and improve the pharmacological properties of tetracycline. Overall, this study establishes a clear relationship between speciation and biological activity in the Zn^2+^–TC system, providing a solid foundation for the rational design of metal–tetracycline complexes as potential anticancer agents.

## Supplementary Material



## Notes

Experimental conditions for UV–vis spectrophotometric, potentiometric, and ^1^H NMR titrations of tetracycline (TC) and Zn^2+^-TC systems (I = 0.15 mol L^–1^, NaCl); hydrolysis constants of Zn^2+^ at different temperatures; calculated chemical shifts for each tetracycline (TC) nucleus; ^1^H NMR spectrum of TC in H_2_O, C_TC_ = 2 mmol L^–1^, t = 25 °C; ^1^H NMR spectrum of TC in deuterated methanol (CD_3_OD) C_TC_ = 2 mmol L^–1^, t = 25 °C; comparison of ^1^H NMR chemical shifts vs pH at t = 25 °C of TC (C_TC_ = 2 mmol L^–1^) and TC in the presence of Zn^2+^ (C_Zn_ = C_TC_ = 2 mmol L^–1^); and comparison of experimental and calculated chemical shifts at t = 25 °C for TC (C_TC_ = 2 mmol L^–1^) and its Zn^2+^ complex (C_Zn_ = C_TC_ = 2 mmol L^–1^) based on ^1^H NMR data (PDF)

## Abbreviations

TC: tetracycline
TCs: tetracyclines
NSCLC: non-small cells lung cancer
DMEM: Dulbecco’s Modified Eagle Medium
FBS: fetal bovine serum
PS: penicillin–streptomycin
PBS: phosphate-buffered saline
DMSO: dimethyl sulfoxide
MTT: 3-(4,5-dimethylthiazol-2-yl)-2,5-diphenyltetrazolium bromide
