# Supplementary material for: Speciation of Tetracycline and Its Zn2+ Complexes in Aqueous Solution and Their Antiproliferative Activity in Non-Small Lung Cancer Cells
Source: ACS Omega. 2026 Jul 17;11(29):44108–17. doi: 10.1021/acsomega.6c04056 (PMC13425761; doi:10.1021/acsomega.6c04056)
Supplement: Supplementary file 1 [file ao6c04056_si_001.pdf]

Speciation of tetracycline and its Zn^{2+} complexes in aqueous solution and their antiproliferative activity in non-small lung cancer cells

Chiara Abate[a], Federica Carnamucio[b,*], Claudia Foti[a], Marsela Ceraj[a], Massimiliano Cordaro[a], Sandro R. P. da Rocha[b], Ottavia Giuffrè[a,*]

[a]*Dipartimento di Scienze Chimiche, Biologiche, Farmaceutiche ed Ambientali, Università di Messina, Viale F. Stagno d'Alcontres 31, 98166 Messina, Italy*.

[b]*Department of Pharmaceutics and Center for Pharmaceutical Engineering and Sciences - School of Pharmacy, Virginia Commonwealth University, 410 N 12th St, 23298, Richmond, Virginia, United States*

[*]carnamuciof@vcu.edu; ogiuffre@unime.it

Supporting Information

Table S1. Experimental conditions for UV-Vis spectrophotometric, potentiometric and ^{1}H NMR titrations of tetracycline (TC) and Zn^{2+}-TC systems (I = 0.15 mol L^{-1}, NaCl).

Technique	t/°C	C_{TC}/mmol L^{-1}	C_{HCl}/mmol L^{-1}	C_M/mmol L^{-1}	M:L	pH range
UV-Vis	15, 25,37,45	0.03	0.12	—	—	3-11
	15, 25,37,45	0.05	0.2	—	—	3-11
	15, 25,37,45	0.075	0.3	—	—	3-11
	15, 25,37,45	0.1	0.4	—	—	3-11
	15, 25,37,45	0.05	0.2	0.05	1:1	3-11
	15, 25,37,45	0.05	0.2	0.025	1:2	3-11
	15, 25,37,45	0.1	0.4	0.033	1:3	3-11
Potentiometry	15, 25,37,45	1	3	1	1:1	2-6
	15, 25,37,45	4	12	2	1:2	2-6
	15, 25,37,45	3	9	1	1:3	2-6
	15, 25,37,45	3	9	1.5	1:2	2-6
^{1}H NMR	25	2	6	—	—	2.3-11.0
	25	2	6	2	1:1	2.2-5.8

Table S2. Hydrolysis constants of Zn^{2+} at different temperatures.

**Reaction**	**t /°C**	**I / mol L^{-1}**	**logβ[1]**
$Zn^{2+} + H_2O = Zn(OH)^+ + H^+$	15	0.15	-9.5
	25	0.15	-9.14
	37	0.15	-8.78
	45	0.15	-10.02[2]
$Zn^{2+} + 2H_2O = Zn(OH)_2^0 + 2H^+$	15	0.15	-17.64
	25	0.15	-17.10
	37	0.15	-16.52
	45	0.15	-16.52[2]
$Zn^{2+} + 3H_2O = Zn(OH)_3^- + 3H^+$	15	0.15	-29.12
	25	0.15	-28.4
	37	0.15	-27.54
	45	0.15	-27.54[2]
$Zn^{2+} + 4H_2O = Zn(OH)_4^{2-} + 4H^+$	15	0.15	-41.67
	25	0.15	-40.40
	37	0.15	-39.47
	45	0.15	-39.47[2]
$2Zn^{2+} + H_2O = Zn_2(OH)^{3+} + H^+$	15	0.15	-9.27
	25	0.15	-8.70
	37	0.15	-8.54
	45	0.15	-8.54[2]
$2Zn^{2+} + 6\ H_2O = Zn_2(OH)_6^{2-} + 6H^+$	15	0.15	-58.91
	25	0.15	-57.50
	37	0.15	-55.9
	45	0.15	-55.9[2]

[1] F. Crea, G. Falcone, C. Foti, O. Giuffrè, S. Materazzi. Thermodynamic data for Pb^{2+} and Zn^{2+} sequestration by biologically important S-donor ligands, at different temperatures and ionic strengths, New J. Chem., 2014, 38, 3973.

[2] K. J. Powell, P. L. Brown, R. H. Byrne, T. Gajda, G. Hefter, A. Leuz, S. Sjöberg, H. Wanner, Chemical speciation of environmentally significant metals with inorganic ligands. Part 5: The Zn^{2+} + $OH^–$, Cl^-, CO_3^{2-}, $SO_4^{2–}$, and $PO_4^{3–}$ systems (IUPAC Technical Report), Pure Appl. Chem., 2013, 85, 2249–2311.

Table S3. Calculated chemical shifts for each tetracycline (TC) nucleus.

Nucleus	δ						
	TC	(TC)H	$(TC)H_2$	$(TC)H_3$	$Zn(TC)H_2$	Zn(TC)H	Zn(TC)
CH(8)	7.254(6) [a)]	7.338(6) [a)]	7.500(6)[a)]	7.506(6) [a)]	7.508(6) [a)]	6.984(6) [a)]	7.494(6) [a)]
CH(7)	6.94(3)	7.01(3)	7.13(3)	7.13(3)	7.14(3)	6.74(3)	7.15(3)
CH(9)	6.70(3)	6.80(3)	6.91(3)	6.91(3)	6.87(3)	7.61(3)	4.84(3)
$CONH_2$	2.71(2)	3.26(2)	3.75(2)	3.98(2)	3.89(2)	3.82(2)	3.49(2)
CH(4a)	2.832(6)	2.911(6)	2.991(6)	3.015(6)	3.048(6)	2.056(6)	5.546(6)
CH_3-N4	2.24(3)	2.615(3)	2.824(3)	2.919(3)	2.7746(3)	3.092(3)	2.669(3)
CH(4)	2.43(3)	2.58(3)	2.66(3)	2.84(3)	2.62(3)	3.02(3)	2.603(3)
CHa(5)	1.99(2)	2.04(2)	2.15(2)	2.17(2)	2.16(2)	1.93(2)	2.20(2)
CHb(5)	1.607(6)	1.692(6)	1.788(6)	1.745(6)	1.775(6)	1.653(6)	1.770(6)
CH_3-C6	1.46(4)	1.47(4)	1.54(4)	1.53(4)	1.55(4)	0.99(4)	1.62(4)

[a)] ≥ 95% interval of confidence.

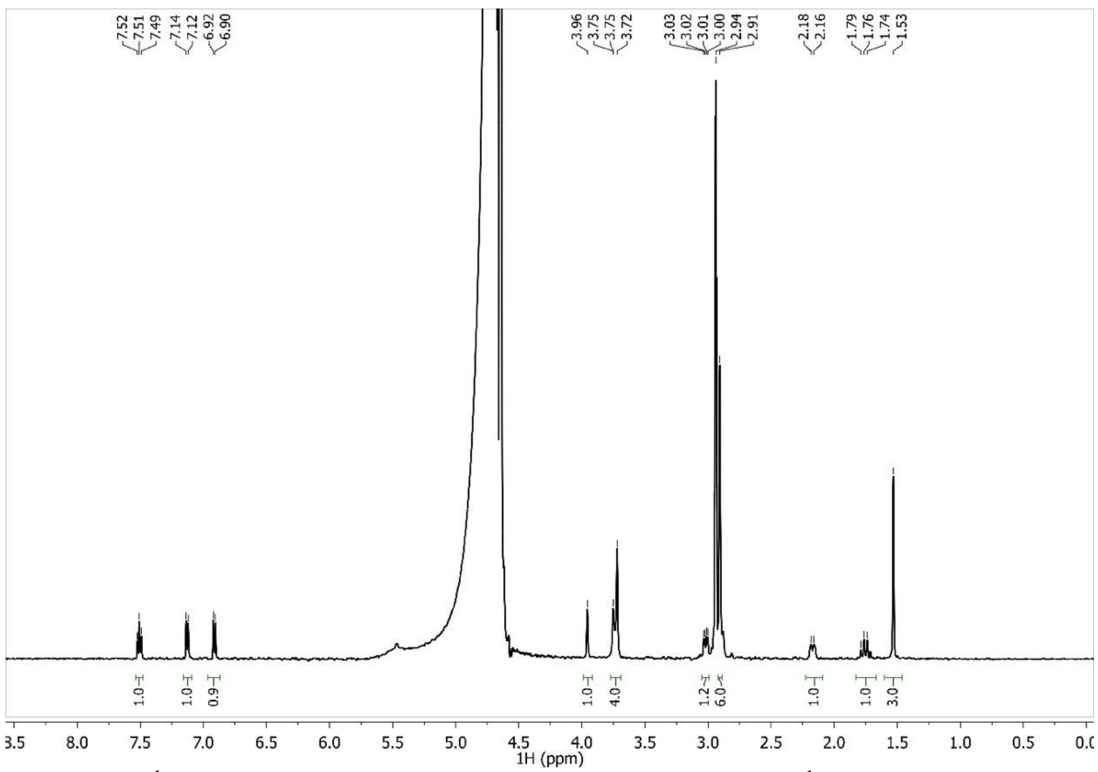

Figure S1. ^{1}H NMR spectrum of TC in H_2O, C_{TC} = 2 mmol L^{-1}, t = 25°C.

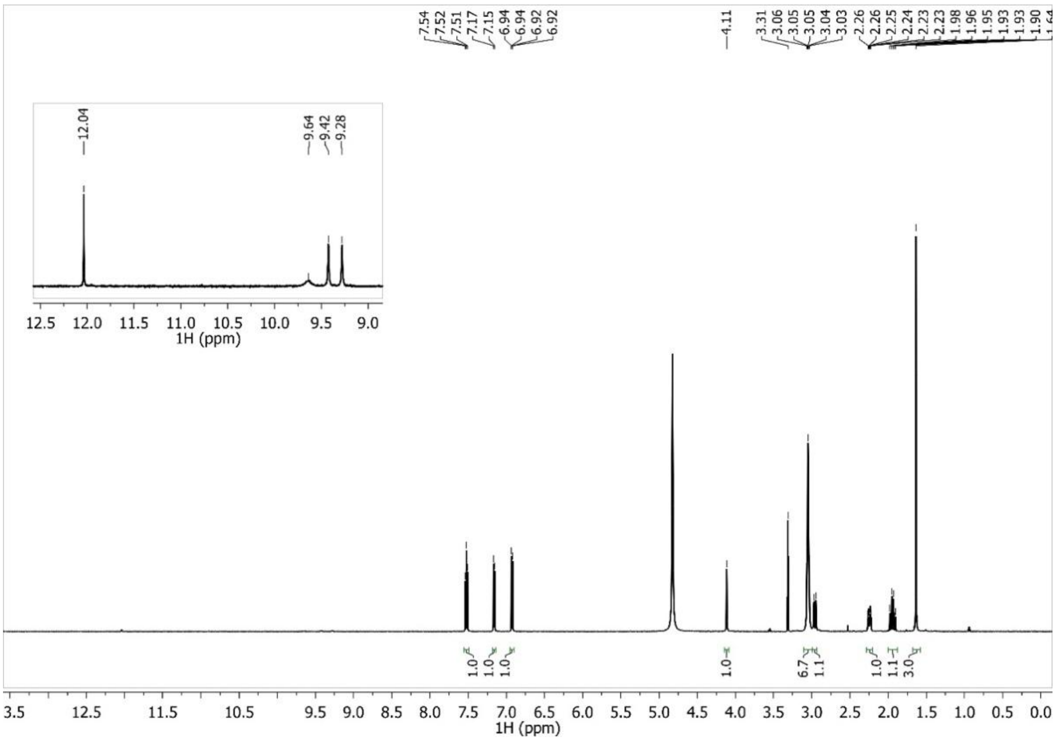

Figure S2. ^{1}H NMR spectrum of TC in deuterated methanol (CD_3OD) C_{TC} = 2 mmol L^{-1}, t = 25°C.

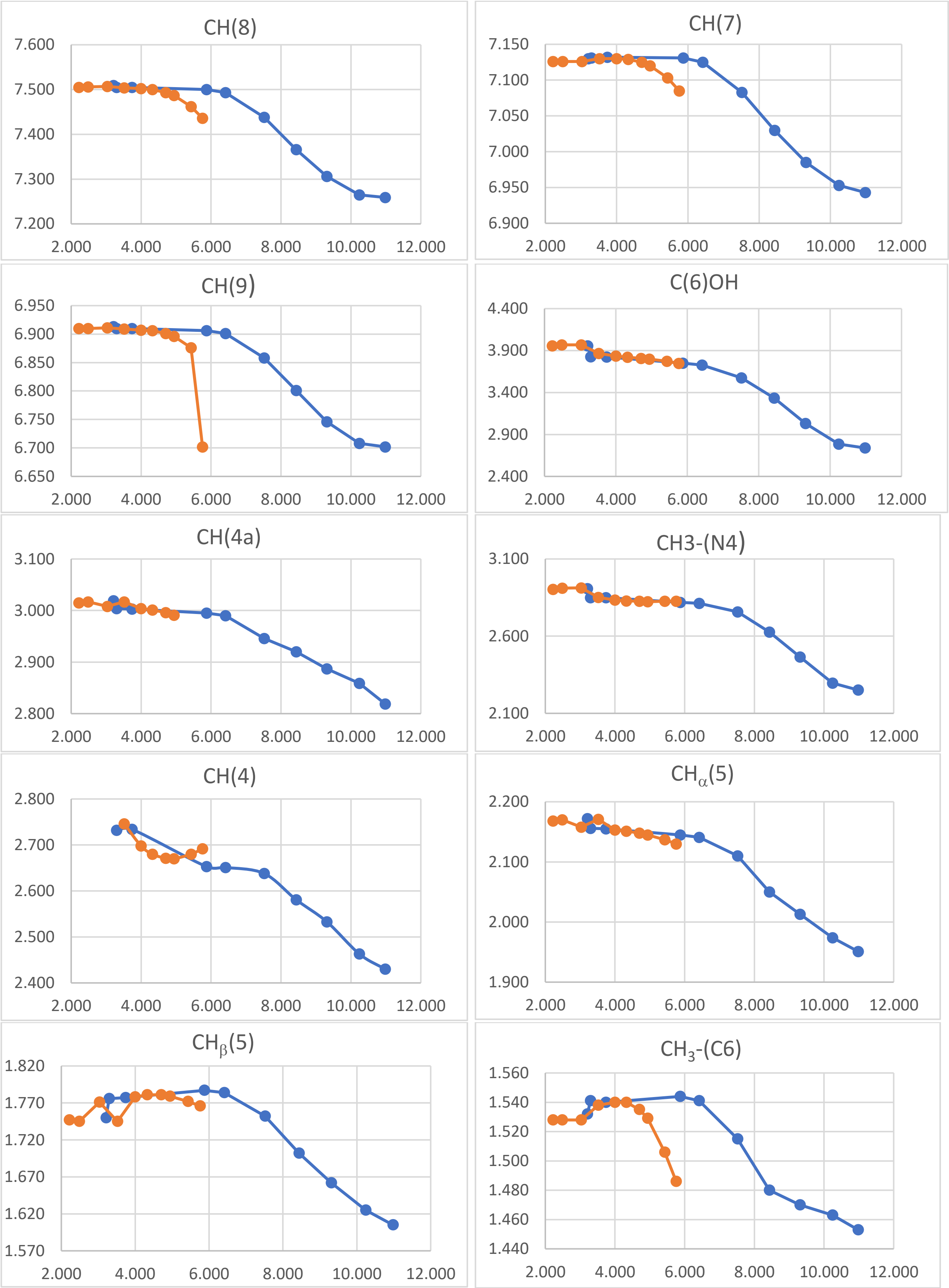

Figure S3. Comparison of ^{1}H NMR chemical shifts vs. pH at t = 25°C of TC (C_{TC} = 2 mmol L^{-1}) and TC in the presence of Zn^{2+} (C_{Zn} = C_{TC} = 2 mmol L^{-1}).

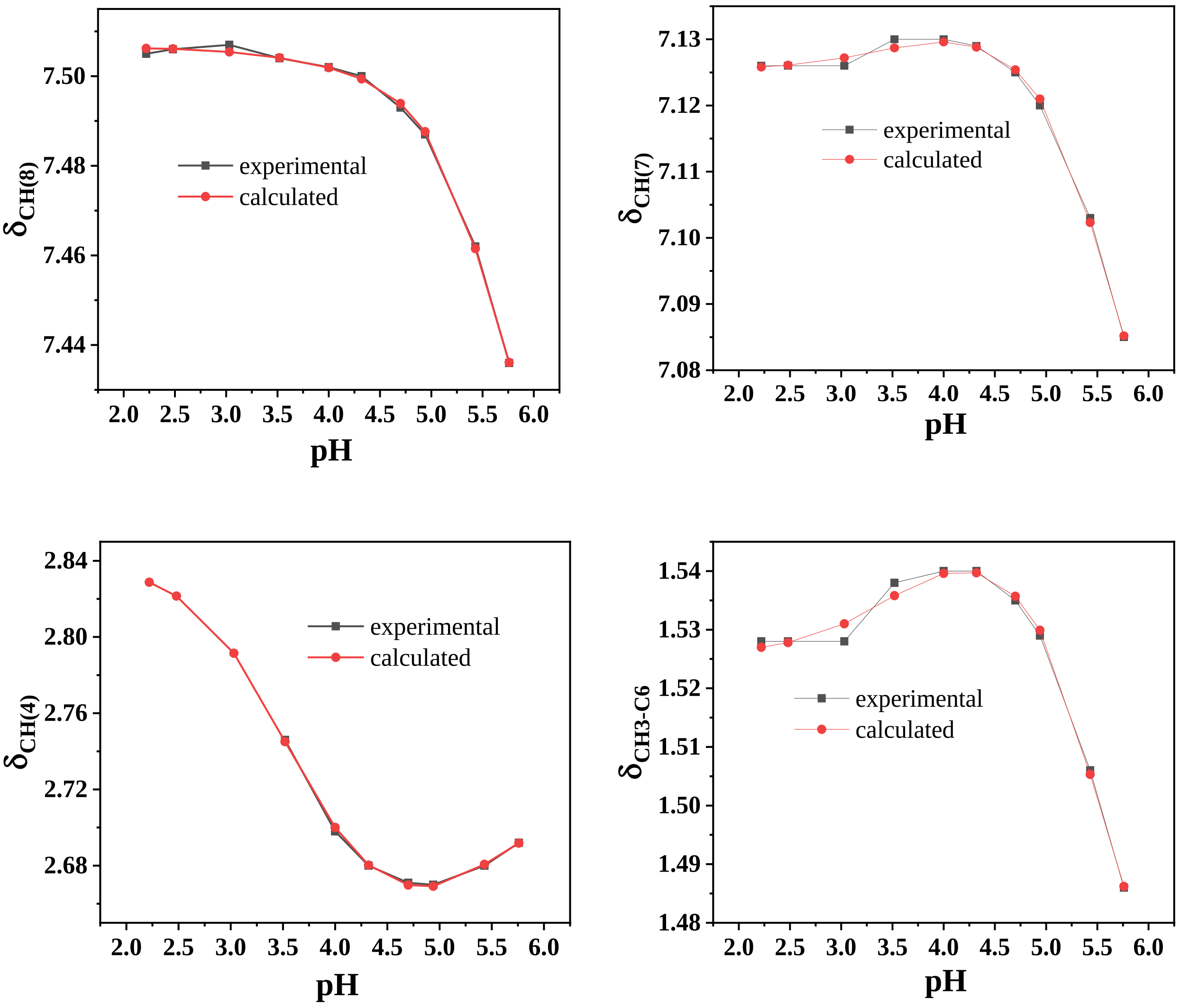

Figure S4. Comparison of experimental and calculated chemical shifts at t = 25°C for TC (C_{TC} = 2 mmol L^{-1}) and its Zn^{2+} complex (C_{Zn} = C_{TC} = 2 mmol L^{-1}) based on ^{1}H NMR data.
